# Optimal breastfeeding durations for HIV‐exposed infants: the impact of maternal ART use, infant mortality and replacement feeding risk

**DOI:** 10.1002/jia2.25107

**Published:** 2018-04-18

**Authors:** Divya Mallampati, Rachel L MacLean, Roger Shapiro, Francois Dabis, Barbara Engelsmann, Kenneth A Freedberg, Valeriane Leroy, Shahin Lockman, Rochelle Walensky, Nigel Rollins, Andrea Ciaranello

**Affiliations:** ^1^ Department of Obstetrics and Gynecology Northwestern University, Chicago IL USA; ^2^ Medical Practice Evaluation Center, Department of Medicine Massachusetts General Hospital and Harvard Medical School Boston MA USA; ^3^ Divisions of General Medicine Department of Medicine Massachusetts General Hospital and Harvard Medical School Boston MA USA; ^4^ Division of Infectious Diseases Beth Israel Deaconess Medical Center Boston MA USA; ^5^ Department of Immunology and Infectious Diseases Harvard T.H Chan School of Public Health Boston MA USA; ^6^ The Botswana–Harvard School of Public Health AIDS Institute Partnership for HIV Research and Education Gaborone Botswana; ^7^ Université Bordeaux Institut de Santé Publique, d'Epidémiologie et de Dévelopement (ISPED), Centre INSERM U1219‐Bordeaux Population Health Bordeaux France; ^8^ Organization for Public Health Interventions and Development Harare Zimbabwe; ^9^ Harvard University Center for AIDS Research Boston MA USA; ^10^ Inserm U1027 University of Toulouse 3 Toulouse France; ^11^ Division of Infectious Disease Brigham and Women's Hospital Boston MA USA; ^12^ Division of Infectious Diseases Department of Medicine Massachusetts General Hospital Harvard Medical School Boston MA USA; ^13^ Department of Maternal Newborn Child and Adolescent Health World Health Organization Geneva Switzerland

**Keywords:** HIV, PMTCT, breastfeeding, modelling, maternal health, infant/child health

## Abstract

**Introduction:**

In 2010, the WHO recommended women living with HIV breastfeed for 12 months while taking antiretroviral therapy (ART) to balance breastfeeding benefits against HIV transmission risks. To inform the 2016 WHO guidelines, we updated prior research on the impact of breastfeeding duration on HIV‐free infant survival (HFS) by incorporating maternal ART duration, infant/child mortality and mother‐to‐child transmission data.

**Methods:**

Using the Cost‐Effectiveness of Preventing AIDS Complications (CEPAC)‐Infant model, we simulated the impact of breastfeeding duration on 24‐month HFS among HIV‐exposed, uninfected infants. We defined “optimal” breastfeeding durations as those maximizing 24‐month HFS. We varied maternal ART duration, mortality rates among breastfed infants/children, and relative risk of mortality associated with replacement feeding (“RRRF”), modelled as a multiplier on all‐cause mortality for replacement‐fed infants/children (range: 1 [no additional risk] to 6). The base‐case simulated RRRF = 3, median infant mortality, and 24‐month maternal ART duration.

**Results:**

In the base‐case, HFS ranged from 83.1% (no breastfeeding) to 90.2% (12‐months breastfeeding). Optimal breastfeeding durations increased with higher RRRF values and longer maternal ART durations, but did not change substantially with variation in infant mortality rates. Optimal breastfeeding durations often exceeded the previous WHO recommendation of 12 months.

**Conclusions:**

In settings with high RRRF and long maternal ART durations, HFS is maximized when mothers breastfeed longer than the previously‐recommended 12 months. In settings with low RRRF or short maternal ART durations, shorter breastfeeding durations optimize HFS. If mothers are supported to use ART for longer periods of time, it is possible to reduce transmission risks and gain the benefits of longer breastfeeding durations.

## 1 Introduction

Infants born to women living with HIV (WLHIV) are at risk for mother‐to‐child HIV transmission (MTCT) through breast milk 1, 2. Although effective, maternal antiretroviral therapy (ART) does not completely prevent transmission 3, 4. This risk can be eliminated by replacement feeding 5, 6, 7; however, in many settings, replacement feeding leads to malnutrition, diarrhoea, and respiratory illness, because infants are exposed to water contaminated with enteric pathogens and fail to receive protective maternal antibodies through breast milk 1, 8. These conditions are leading causes of infant and under‐five mortality in sub‐Saharan Africa and may account for up to 45% of deaths in children under 5 worldwide 9. In sub‐Saharan Africa, breastfeeding for durations greater than 12 months is common 10. Recommendations and practices among women with HIV vary widely, with an estimated 10‐57% of WLHIV avoiding breastfeeding altogether 5, 6, 7, 11.

To achieve this balance between the risk of HIV transmission and the risk of replacement feeding, the 2010 World Health Organization (WHO) Guidelines on HIV and Infant Feeding defined a primary goal of maximizing infant HIV‐free survival (HFS) 1. These guidelines, using two modelling analyses that outlined optimal feeding strategies as well as the cost to healthcare systems, advised that, in settings where diarrhoea, pneumonia and malnutrition are prevalent, national authorities should support mothers living with HIV whose infants were either uninfected or had an unknown status to exclusively breastfeed for the first 6 months of life, then to continue breastfeeding with complementary foods through 12 months while providing ART to mothers 1. In previous work, we used the Cost‐effectiveness of Preventing AIDS Complications (CEPAC)‐Infant model to examine breastfeeding durations that maximized HFS under various conditions 12. That analysis demonstrated the potential benefit of tailoring breastfeeding durations to maternal CD4 counts, ART availability, and mortality risk associated with replacement feeding; we concluded, however, that recommending 12 months was appropriate in resource‐limited settings as the gains from an “individualized approach” were small. New data have since emerged regarding MTCT risk as a function of maternal retention in care and adherence to ART 13, 14. We incorporated these new data and refined our analysis on the impact of context‐specific infant mortality rates and maternal ART use on optimal feeding recommendations. In 2016, in order to accommodate local breastfeeding practices that extended past 12 months and to harmonize infant feeding recommendations for women living with and without HIV, the WHO considered increasing the recommended duration of breastfeeding for women on ART from 12 to 24 months. This analysis contributed to the updated WHO guidelines on infant feeding and HIV 15.

## 2 Methods

### 2.1 Analytic overview

We used the CEPAC‐Infant model to simulate HIV‐exposed, uninfected infants through 24 months of life 12, 16. The primary outcome was 24‐month HFS, defined as the proportion of the cohort that was alive and uninfected at 24 months of life. Building upon our prior analysis, we incorporated updated MTCT data for women on ART, as well as updated neonatal, infant and under‐five mortality rates from 21 Global Plan priority countries 2, 5, 12, 13, 14, 17, 18, 19, 20, 21, 22, 23, 24, 25, 26, 27, 28, 29. We projected HFS for each breastfeeding duration from 0 (replacement‐fed from birth) through 24 months, while simultaneously varying three key parameters: the duration of maternal ART, the relative risk of infant and child mortality associated with replacement feeding compared to breastfeeding (RRRF), and background infant and child mortality rates. RRRF was modelled as a multiplier on monthly background breastfed mortality risks, applied during each month infants were replacement fed. The modelled duration of maternal ART use was intended to reflect both retention in care and ART adherence 13. The primary goals of our analysis were: (1) to determine the “optimal breastfeeding duration” or the duration that maximized 24‐month HFS for each scenario, and (2) to quantify the impact of maternal ART duration on 24‐month HFS for each combination of RRRF and breastfeeding duration. In the base case, we simulated 24 months of maternal ART, an RRRF of 3, and median infant/child mortality rates. In sensitivity analyses, we varied each of these parameters; in secondary analyses, we varied monthly MTCT rates and examined variations in RRRF with infant/child age.

### 2.2 CEPAC model and structure

#### 2.2.1 CEPAC‐infant model

The CEPAC‐Infant model is a Monte Carlo microsimulation model of infant HIV infection and survival from birth through 24 months of age. The CEPAC‐Infant model is simpler in structure than the CEPAC‐Adult and CEPAC‐Paediatric models (Figure S1) 12, 30. In the CEPAC‐Infant model, HIV‐exposed, uninfected infants enter the model at birth and face three key monthly risks: (1) background mortality risks for breastfed children, which are multiplied by the RRRF for replacement‐fed children; (2) postnatal HIV infection (MTCT) risk throughout the breastfeeding period, stratified by maternal ART use and maternal CD4 count for women not on ART; and (3) maternal mortality, which leads a child to wean immediately and face the mortality risks of replacement feeding (RRRF). The primary model outcome is monthly HFS: the proportion of children alive and HIV‐uninfected at each month after birth; the model has been calibrated to data from sub‐Saharan Africa 16, 31, 32. Furthermore details about the full CEPAC‐Infant model can be found in the technical appendices of prior CEPAC‐Infant analyses 12, 30 as well as at http://web2.research.partners.org/cepac/model.php.

Because traditional measures of statistical significance (*p*‐values, 95% confidence intervals) do not accurately reflect precision in microsimulation model results, we instead follow International Society for Pharmacoeconomics and Outcomes Research/Society for Medical Decision Making (ISPOR/SMDM) guidance and describe the impact of uncertainty in our analysis through a series of extensive univariate and multivariate sensitivity analyses 33. A full description of our approach to 16 specific ISPOR/SMDM recommendations for analysing uncertainty in the CEPAC model is also provided in the Appendix of previous work 30.

### 2.3 Input parameters

#### 2.3.1 Breastfeeding duration

We modelled breastfeeding durations of 0 to 24 months in 3‐month increments. Infants assigned to breastfeed for 0 months initiated replacement feeding immediately after birth. For all others, infants were assumed to wean immediately after the last month of breastfeeding. Breastfeeding in the first 6 months of life was modelled as exclusive breastfeeding, per WHO guidelines, and breastfeeding after 6 months of age was modelled as complementary feeding, consisting of breast milk and food 1.

#### 2.3.2 Maternal ART duration

In the base case, we assumed that women took ART throughout the simulation (24 months), reflecting WHO “Option B+” guidelines for lifelong ART. In sensitivity analyses, we varied maternal ART duration from 0 to 24 months in 3‐month increments. Because maternal ART duration is not directly specified in the CEPAC‐Infant model structure, we adjusted model output to reflect the impact of maternal ART cessation through a series of recalculated monthly HFS risks (Material S2). We assume that MTCT risk after maternal ART cessation returns immediately to that of a mother who never received ART.

#### 2.3.3 Relative risk of replacement feeding (RRRF)

In the model, RRRF is applied as a multiplier on monthly all‐cause infant/child mortality risk during each month children are replacement fed. We designed the RRRF to reflect the combined impact of replacement feeding on mortality, including effects of poor water quality, insufficient quantities of replacement milk, diarrhoeal disease, or pneumonia. Based on available data, RRRF was not applied during months of complementary feeding. This simulates the protective benefit of breastfeeding throughout the period of breast milk exposure; as seen in previous work, the opposite assumption – ending the protective effect of breastfeeding at 6 months of age – leads all optimal breastfeeding durations for women on ART to be 6 months 3, 5, 6, 7, 12, 34, 35, 36, 37, 38. In the base case, we modelled an RRRF of 3, a value reported in settings like Malawi and Zambia (8, 32 to 33); in sensitivity analyses, this value ranged from 1 to 6, reflecting data from various settings (Table 1) 3, 5, 6, 7, 34, 35, 36, 37, 38.

**Table 1:** Selected model input parameters for the CEPAC‐Infant model

Parameter	Value	Reference
I. Relative risk of mortality among replacement‐fed compared to breastfed infants (RRRF), by country^a^		
Kenya; Rwanda; South Africa; Côte d'Ivoire	1.0	3, 5, 6, 7
Botswana	2.0	34
Malawi	1.8‐3.3	35
Zambia	2.0‐4.2	8, 36
Uganda	6.0	38
II. Monthly infant mortality risk, by scenario and age^b^		49, 50
Moderate mortality (base case)		
0 to 2 months	0.0116	
3 to 11 months	0.0024	
12 to 24 months	0.00067	
Low mortality scenario		
0 to 2 months	0.0082	
3 to 11 months	0.0023	
12 to 24 months	0.0004	
High mortality scenario		
0 to 2 months	0.0172	
3 to 11 months	0.0034	
12 to 24 months	0.00093	
III. Monthly maternal mortality risk, by disease status and ART strategy		Projected from CEPAC‐International adult model
CD4 ≤ 350/μL		
No ART	0.0078	
3‐drug ART	0.0016	
CD4 > 350/μL		
No ART	0.0024	
3‐drug ART	0.0009	
IV. Monthly postnatal mother‐to‐child transmission risk in postnatal period, by model and ART strategy		
Base‐case		
No ART	0.0044	2, 19, 20, 22, 23, 27, 28, 29, 34, 57
3‐drug ART	0.0019	2, 19, 20, 22, 23, 27, 28, 29, 34, 57
Spectrum 2012 inputs		
No ART	0.0095	58
3‐drug ART	0.0020	58
Spectrum 2016 inputs (proposed)		
No ART	0.0108	Personal communication with Spectrum team
3‐drug ART	0.0012	Personal communication with Spectrum team

a RRRF was calculated by dividing cumulative mortality risk (at the greatest duration reported in each study) among replacement‐fed infants by cumulative mortality risk among breastfed infants.

b Mortality rates are stratified by current age in each month of the simulation and reported for breastfed, HIV‐exposed, uninfected children.

In secondary analyses, we evaluated extreme RRRF values (up to 23, far higher than reported in any setting) to identify the threshold at which replacement feeding conferred such high risks that all infants, regardless of maternal ART duration, would need 24 months of breastfeeding to optimize HFS 39. We also examined scenarios in which RRRF was varied with infant/child age, based on limited data from HIV‐unexposed children suggesting that mortality risks of replacement feeding may decrease as an infant ages 40, 41. In this analysis, we decreased the at‐birth RRRF by 50% at 12 months of age, and then by another 50% at 18 months.

#### 2.3.4 Mortality risks

To better reflect variations in infant‐mortality across a range of countries, at WHO request, we used age‐specific monthly infant/child mortality risks derived from published neonatal, infant, and under‐5 mortality rates from 21 Global Plan countries in sub‐Saharan Africa (Table 1) 18. Because breastfeeding is very common in most of the countries from which these data were derived, we have assigned these risks as the “background” mortality risk among breastfed infants; we vary these rates widely in sensitivity analyses. The median risks from all 21 countries were used in the base case analysis (a “moderate” mortality scenario). Because the RRRF and underlying background mortality rates may vary independently, we also evaluated a “low” mortality scenario and a “high” mortality scenario for our sensitivity analyses, using the 25th and 75th percentile values. Monthly maternal mortality risks were derived from previous CEPAC‐Adult modelling analyses for postpartum women (28) (Table 1).

#### 2.3.5 Mother‐to‐child transmission risks

We derived MTCT risks from published trials in breastfeeding African women (Table 1). For infants whose mothers were not on ART, we weighted CD4‐stratified MTCT risks according to the CD4 distribution observed in the representative MTCT‐Plus cohort 17. For infants of mothers on effective ART, maternal CD4 did not impact modelled MTCT risk 2, 5, 19, 20, 21, 22, 23, 24, 25, 26, 27, 28, 29. In sensitivity analyses, we repeated all analyses with MTCT risks derived by the UNAIDS Reference Group for the Spectrum model in 2012 42, as well as proposed Spectrum inputs for 2016 (Nigel Rollins, personal communication); compared to base case inputs, both sets of Spectrum inputs, derived via an independent literature review, assigned slightly higher MTCT risks for women not on ART, and were similar for women on ART.

## 3 Results

### 3.1 Base case results: impact of breastfeeding duration

**Figure 1:**
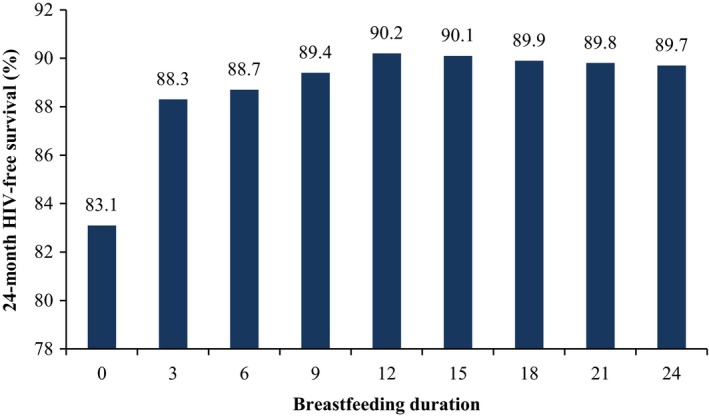
HIV‐free survival by breastfeeding duration for HIV‐exposed infants with moderate infant mortality, RRRF = 3, and maternal ART use throughout the breastfeeding period (base case). Breastfeeding duration is shown on the horizontal axis, and HIV‐free survival is shown on the vertical axis, as well as above the bar for each breastfeeding duration. RRRF, relative risk of replacement feeding; BF, breastfeeding; ART, antiretroviral therapy.

In the base case (RRRF=3, median infant mortality, maternal ART duration=24 months), 24‐month HFS ranged from 83.1% with replacement feeding from birth to 90.2% with breastfeeding duration of 12 months (Figure 1). Although breastfeeding for 12 months maximized HFS, breastfeeding durations ≥12 months yielded similar HFS values (89.7% to 90.2%), suggesting that a change from 12 to 24 months of breastfeeding would have minimal impact on HFS.

### 3.2 Sensitivity analyses

#### 3.2.1 Maternal ART duration

The impact of varying maternal ART duration is shown in Table 2, section I. The base‐case (24‐month ART duration) is shown on the far right (box). The sections to the left vary breastfeeding and maternal ART duration, holding constant the RRRF (set at 3) and infant mortality (median values). For example, when the breastfeeding duration is 12 months as in the base case (bold), HFS remained 90.2% as long as maternal ART duration was also ≥12 months (yellow). For any given maternal ART duration from 3 to 12 months, HFS was usually maximized by breastfeeding for as long as maternal ART was taken, for example, with 9‐months maternal ART, by breastfeeding for 9 months (HFS 89.4%, yellow). For any given breastfeeding duration beyond 0, HFS increased substantially if maternal ART use persisted throughout the entire breastfeeding period; with 24 months of breastfeeding, for example, HFS increased from 74.1% with no ART to 89.7% with 24 months of ART (bottom row).

**Table 2:** 24‐month HIV‐free survival for selected breastfeeding durations, maternal ART durations, and RRRF values with moderate mortality risk (full results in Supplemental 635 Table S3)

I RRRF = 3									
RR‐RF = 3	24‐month HIV‐free survival (%)								
ART duration **→**	**0**	**3**	**6**	**9**	**12**	**15**	**18**	**21**	**24**
BF 0 months	83.1	83.1	83.1	83.1	83.1	83.1	83.1	83.1	83.1
BF 3 months	87.5	88.3	88.3	88.3	88.3	88.3	88.3	88.3	88.3
BF 6 months	87.3	88	88.7	88.7	88.7	88.7	88.7	88.7	88.7
BF 9 months	85.3	86	86.7	89.4	89.4	89.4	89.4	89.4	89.4
BF 12 months	**83.4**	**84.1**	**84.7**	**87.4**	**90.2**	**90.2**	**90.2**	**90.2**	**90.2**
BF 15 months	80.9	81.6	82.2	84.8	87.5	90.1	90.1	90.1	90.1
BF 18 months	78.5	79.2	79.8	82.3	84.9	87.4	89.9	89.9	89.9
BF 21 months	76.2	76.9	77.4	79.9	82.4	84.9	87.3	89.8	89.8
BF 24 months	74.1	74.8	75.3	77.7	80.2	82.6	84.9	87.3	89.7

II RR‐RF = 1									
RR‐RF = 1	24‐month HIV‐free survival (%)								
ART duration **→**	**0**	**3**	**6**	**9**	**12**	**15**	**18**	**21**	**24**
BF 0 months	93.8	93.8	93.8	93.8	93.8	93.8	93.8	93.8	93.8
BF 3 months	92.6	93.3	93.3	93.2	93.3	93.3	93.3	93.3	93.3
BF 6 months	91.4	92.1	92.7	92.7	92.7	92.7	92.7	92.7	92.7
BF 9 months	88.2	88.2	89.4	92.2	92.2	92.2	92.2	92.2	92.2
BF 12 months	**85.1**	**85.7**	**86.3**	**89**	**91.7**	**91.7**	**91.7**	**91.7**	**91.7**
BF 15 months	82.2	82.9	83.4	86	88.6	91.2	91.2	91.2	91.2
BF 18 months	79.6	80.1	80.7	83.2	85.7	88.2	90.7	90.7	90.7
BF 21 months	77	77.6	78.1	80.6	83	85.4	87.8	90.2	90.2
BF 24 months	74.7	75.2	75.7	78.1	80.4	82.8	85.1	87.4	89.7

III RRRF = 6									
RR‐RF = 6	24‐month HIV‐free survival (%)								
ART duration →	**0**	**3**	**6**	**9**	**12**	**15**	**18**	**21**	**24**
BF 0 months	69.4	69.3	69.3	69.3	69.3	69.3	69.3	69.3	69.3
BF 3 months	80.5	81.3	81.3	81.3	81.3	81.3	81.3	81.3	81.3
BF 6 months	81.4	82.2	82.9	82.9	82.9	82.9	82.9	82.9	82.9
BF 9 months	81.2	82	82.7	85.4	85.4	85.4	85.4	85.4	85.4
BF 12 months	**81**	**81.8**	**82.5**	**85.2**	**88**	**88**	**88**	**88**	**88**
BF 15 months	79	79.7	80.4	83	85.8	88.4	88.4	88.4	88.4
BF 18 months	77	77.7	78.4	81	83.7	86.2	88.8	88.8	88.8
BF 21 months	75.1	75.9	76.5	79	81.7	84.1	86.7	89.2	89.2
BF 24 months	73.4	74.1	74.8	77.2	79.8	82.2	84.7	87.1	89.6

ART, antiretroviral therapy; BF, breastfeeding duration; RRRF, relative risk of replacement feeding; HFS, HIV‐free survival.

Bolded numbers indicate HFS at 12 months (2010 WHO Recommendation). Red box indicates base case scenario (RRRF = 3, 24‐month ART duration, median infant/child mortality). Yellow indicates maximum HFS for each ARV duration.

#### 3.2.2 Relative risk of replacement feeding

The impact of varying Relative risk of replacement feeding (RRRF) is shown in Table 2, sections II and III, which report results for RRRF = 1 and RRRF = 6 (results for all RRRF values are in Table S3). At an RRRF of 1 (no additional mortality risk from replacement feeding), the optimal breastfeeding duration was 0 months regardless of maternal ART duration, with a 24‐month HFS of 93.8% (Section II, yellow). For all RRRF values >1, HFS was maximized by longer breastfeeding durations as RRRF increased. At a maternal ART duration of 24 months, HFS was maximized by 12 months breastfeeding if RRRF = 3 (base case, Section I, box and yellow, HFS 90.2%) and by 24 months breastfeeding if RRRF = 6 (Section III, yellow, HFS 89.6%).

**Figure 2:**
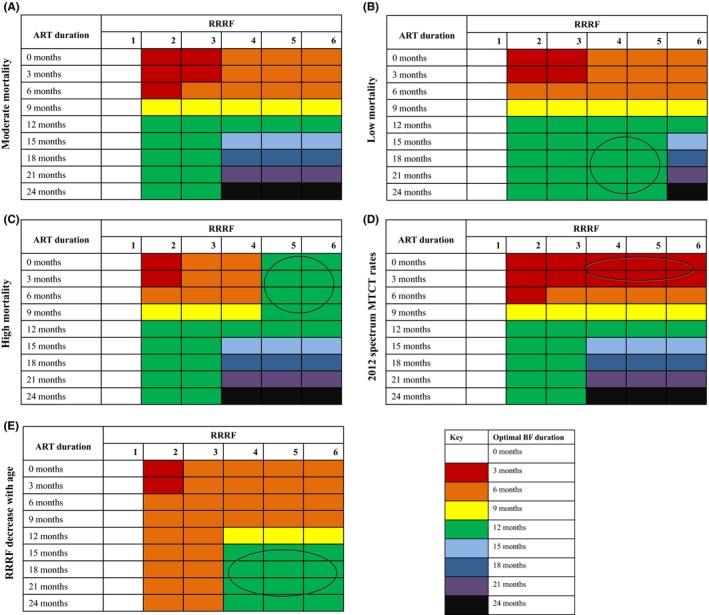
Optimal breastfeeding duration for HIV‐exposed infants by RRRF value and maternal ART duration in various settings. This figure displays the breastfeeding duration that optimizes HIV‐free survival for each combination of RRRF value and ART duration. RRRF value varies across the horizontal axis, and maternal ART duration varies across the vertical axis. Colours correspond to different breastfeeding durations, as described in the figure key. Circles indicate differences in optimal breastfeeding duration compared to the base case. **(A)** Results for “moderate mortality” scenario. **(B)** Results for “low mortality” scenario, using 25th percentile values of neonatal, infant, and under‐five mortality estimates from 21 Global Plan countries. **(C)** Results for “high mortality” scenario, using 75th percentile values of neonatal, infant, and under‐five mortality estimates from 21 Global Plan countries. **(D)** Results using MTCT rates used in the 2012 Spectrum model. Results using Spectrum 2016 rates are similar and are shown in Material S5. **(E)** Results for scenarios in which RRRF is reduced as children age. RRRF impact was reduced by 50% for infants who reach 12 months of age, and was again reduced by 50% for infants at 18 months of age. RRRF, relative risk of replacement feeding; BF, breastfeeding; ART, antiretroviral therapy.

The interaction between RRRF and maternal ART duration affected optimal breastfeeding duration (Figure 2A; Table S3). At low RRRF values (e.g. 2 to 3), HFS was maximized by weaning at 12 months (green) even if mothers continued on ART thereafter (and by breastfeeding for at least 3 months (red) even if ART was stopped sooner). At higher RRRF values (e.g. 4 to 6), HFS was maximized by breastfeeding for as long as maternal ART continued (and for at least 6 months at RRRF ≥4 (orange), even if ART was stopped sooner).

#### 3.2.3 Mortality risks

With low infant/child mortality risks, the only difference from the base case was seen at RRRF values of 4 to 5, where optimal breastfeeding durations were shorter than in the base case (Figure 2B, circle). In this scenario, optimal breastfeeding duration only exceeded 12 months if RRRF = 6 and maternal ART duration was also >12 months (blue through black). With high infant/child mortality (Figure 2C), the only difference from the base case occurred at high RRRF values and short ART durations (RRRF = 5 to 6 and ART durations ≤9 months; circle): here, optimal breastfeeding durations were longer than the base case, for example, at least 12 months (green) regardless of maternal ART duration.

### 3.3 Secondary analyses

#### 3.3.1 Mother‐to‐child transmission risks

In most scenarios, use of 2012 Spectrum MTCT risk inputs resulted in optimal breastfeeding durations similar to the base case (Figure 2D). There were exceptions at higher RRRF values (≥4) when women were on ART for short durations (0 to 3 months), in these cases, the optimal breastfeeding duration was shorter than in the base case. This reflected Spectrum estimates that had higher off‐ART transmission risks and thus favoured shorter breastfeeding durations (Panel D, circle). Results with proposed 2016 Spectrum inputs were similar to those with 2012 inputs (Figure S5).

#### 3.3.2 RRRF: Extreme values and changes with age

When RRRF declined as infants aged, the optimal breastfeeding durations were shorter than in the base case, and only exceeded 6 months if initial RRRF was ≥4 and maternal ART duration was ≥12 months (Figure 2E, yellow/green, circle). In order for the optimal breastfeeding duration to be 24 months, regardless of maternal ART duration, an RRRF value of ≥21 was needed (Material S4B, black).

## 4 Discussion

We used the CEPAC‐Infant model to identify the breastfeeding duration that maximizes HFS for children born to mothers living with HIV under varying conditions of infant/child mortality, replacement feeding risks and maternal ART duration. There are three primary findings from this work.

First, as mortality risks associated with replacement feeding increase, optimal breastfeeding durations increase; survival with breastfeeding begins to outweigh HIV transmission risks. In the special case of RRRF = 1, breastfeeding does not confer a mortality benefit and leads only to HIV transmission risk. For all RRRF values >1, however, optimal breastfeeding duration increases as RRRF increases. These findings are unchanged in most of the scenarios we examined. Key exceptions are lower MTCT risks for women not on ART (Spectrum inputs, influential only at high RRRF values and short ART durations); higher or lower infant mortality rates (minimally influential only at high RRRF values); and the assumption that RRRF declines with infant age (leading to shorter optimal breastfeeding durations in all scenarios when RRRF at birth is ≥2).

Second, in many scenarios, 24‐month HFS is maximized by breastfeeding durations longer than the previously WHO‐recommended 12 months. This is particularly true when RRRF values are high and maternal ART is taken throughout breastfeeding. In most scenarios, however, the difference in HFS that would result from shortening or extending breastfeeding to “optimal” durations is minimal (<1%). Similarly, several country‐specific examples favor slightly shorter breastfeeding durations. For example, Rwanda, with an RRRF value nearing 1 and low mortality rates, would have an optimal breastfeeding duration of 0 months 5. In contrast, Malawi has a moderate neonatal and infant mortality rate, yet has RRRF values near 3; with high PMTCT coverage, the optimal breastfeeding duration would be close to 6 months 35. A decision to recommend 12 *versus* 24 months of breastfeeding could reasonably be based on several factors, such as the important goal of harmonizing with recommendations for HIV‐uninfected women or local practices with regard to breastfeeding duration.

Third, continuation of maternal ART throughout breastfeeding is critical to maximizing child HFS, particularly if RRRF is low. For any given RRRF value and breastfeeding duration of at least 6 months, HFS is maximized when maternal ART is continued at least through the time of weaning. Breastfeeding for 24 months in the absence of long‐term maternal ART is only optimal when RRRF values are implausibly high (≥21). Supporting ART use throughout breastfeeding may be difficult; during the postpartum period, many women face medication stockouts, adherence challenges, or are lost from care 43. In those situations, however, weaning is not the answer: reliable access to replacement foods and clean water are likely to be equally challenging. Even if countries were to prioritize safe water supplies, and thus reduce their RRRF values and shorten optimal breastfeeding durations, formula can often be expensive and inaccessible for mothers and their infants. This analysis demonstrates the importance of supporting women to remain in care and on ART through breastfeeding 13. In addition, although child HFS is not affected by maternal ART use after weaning, there are substantial benefits for maternal health, and for prevention of HIV transmission to sexual partners and future children, when women remain on ART 44, 45, 46, 47.

There are several limitations to our analysis. First, we used country‐specific infant/child mortality rates from the general population, which are lower than mortality rates reported among HIV‐exposed, uninfected (HEU) infants used in our prior analysis 48, 49, 50. After adjusting for feeding status and maternal health, however, it is not known if HEU infants have higher mortality than the general population. If mortality rates among HEU infants are truly closer to those in our “high” mortality scenario, optimal breastfeeding durations will nearly always exceed 12 months. Moreover, we used “background” mortality risks to reflect the mortality rates among breastfed infants; if the true rates among breastfed infants are lower than the rates we used, our results of optimal breastfeeding durations in our base case scenario would more closely resemble a “low mortality” scenario and thus possibly favor shorter optimal breastfeeding durations. Second, our model structure requires that MTCT risks after ART discontinuation immediately equal those for women never on ART. Although there are no data on how quickly MTCT risks rise when ART is interrupted, this assumption likely overestimates MTCT risks in the months immediately after ART discontinuation. If these risks were lower, optimal breastfeeding durations would be slightly longer than reported here. Third, this analysis only examines ART duration. Our model assumes that once a woman stops taking ART, she does not reinitiate ART during breastfeeding. Unlike our prior analysis, we do not explicitly vary access or adherence to ART for women retained in care, which may fade during the postpartum period 13. Reduced ART access or adherence would increase MTCT risks and favor shorter breastfeeding durations 12. Fourth, the WHO‐recommended outcome of HIV‐free survival gives equal “importance” to HIV infection and death 1. As ART access for HIV‐infected children improves, leading to longer survival and improved quality of life, this equal weighting becomes less appropriate. Fourth, our analysis excludes costs. Several previous analyses have reported cost savings associated with breastfeeding compared to replacement feeding; however, these have primarily focused on the cost of formula milk and excluded the healthcare resources required to care for diarrhoeal disease, pneumonia, malnutrition, or HIV disease 51, 52. Including these costs could be important for understanding the programmatic requirements and feasibility of supporting either replacement or breastfeeding.

Although the RRRF value reflects a critical concept in the balance of risks and benefits needed to inform infant feeding decisions, data to inform this parameter are limited. We derived values from published research, and it may be difficult for countries or communities to predict their own RRRF values. We anticipate that most settings in sub‐Saharan Africa will have RRRF values of 2 to 3 3, 5, 6, 7, 34, 35, 36, 37, 38. Sites with good‐quality water or with comprehensive access to healthcare services for infants, may have RRRF values close to 1 7, while specific situations such as diarrhoeal outbreaks may be associated with RRRF values of 6 or greater 34, 39. Improved data on variation in replacement feeding risks over time are also needed. One analysis has reported a decrease in RRRF over time, but these data were derived from HIV‐unexposed infants outside of Africa, so their generalizability to HIV‐exposed sub‐Saharan African infants is unknown 40. If RRRF decreases as children age, we find that optimal breastfeeding only exceeds 6 months with high at‐birth RRRF (≥4) and long maternal ART duration (≥12 months).

In conclusion, this analysis provides new information regarding the impact of infant mortality, replacement feeding risks, and maternal ART duration on the feeding decisions that optimize HFS among HIV‐exposed infants. Our findings extend those of prior model‐based reports, which simulated MTCT in the absence of maternal or infant ART and examined a limited number of breastfeeding durations 53, 54, 55, 56. Like our previous analysis, this paper similarly finds that an individualized approach to identify optimal breastfeeding durations can maximize infant HFS, but confers only modest gains compared to a public health approach that suggests the same breastfeeding duration for all women with HIV taking ART 12. The current analysis, however, additionally evaluates the balance between replacement feeding risks, new information regarding ART regimens during breastfeeding and associated transmission risks, and emerging data about ART adherence and retention postpartum. Our findings in infant/child mortality risks and RRRF (as a proxy for water safety and formula access), may be used to examine our results as applicable in a wide range of settings. In many settings, breastfeeding durations longer than the 2010 WHO recommendation of 12 months would maximize child 24‐month HFS, although in settings where mortality risks associated with replacement feeding are very low, optimal breastfeeding durations are shorter than those recommendations. Importantly, however, the differences in HFS between “optimal” and recommended breastfeeding durations are small in most cases. Adherence to maternal ART throughout the entire breastfeeding period improves HFS far more than the specific breastfeeding duration itself and also leads to benefits for mothers, their partners, and their subsequent children. These findings directly informed new WHO guidance about infant feeding 15, and emphasize that programmes should focus on supporting mothers to remain in care and on ART for life, both for their own health and for the health of their children.

### 4.1 Study period

This analysis was performed between July and December 2015.

## Competing interests

The authors declare no conflicts of interest. The content is solely the responsibility of the authors and does not necessarily represent the official views of the World Health Organization.

## Authors’ Contributions

DM, RLL and AC were responsible for the design of this research as well as the writing/revision of this manuscript. RS, FD, BE, KAF, VL, SL, RW and NR were instrumental in the writing and revision of this manuscript.

## Supporting information


**Appendix S1**. Additional methods, tables, and figures.
