# Supplementary material for: Optimal breastfeeding durations for HIV‐exposed infants: the impact of maternal ART use, infant mortality and replacement feeding risk
Source: J Int AIDS Soc. 2018 Apr 18;21(4):e25107. doi: 10.1002/jia2.25107 (PMC5904528; doi:10.1002/jia2.25107)

**An Analysis of Optimal Breastfeeding Durations for HIV-Exposed Infants: Assessing the Impact of Maternal ART Use, Infant Mortality, and Replacement Feeding Risk**

**Supplementary Material**

Divya Mallampati

*et. al*

**Supplementary Material**

This supplementary appendix contains additional methods, tables, and figures to supplement the main text.

**Supplementary Material 1:** Structure of the CEPAC Infant Model

**Supplementary Material 2:** HIV-free survival (HFS) calculations by maternal antiretroviral therapy (ART) duration

**Supplementary Material 3**: 24-month HFS as breastfeeding duration, maternal ART duration, and relative risk of mortality associated with replacement feeding (RRRF) are varied

**Supplementary Material 4A**: 24-month HIV-free survival at the optimal breastfeeding duration at high RRRF values, by maternal ART duration

**Supplementary Material 4B:** Optimal breastfeeding durations at high RRRF values, by maternal ART duration

**Supplementary Material 4C**: Difference in 24-month HIV-free survival between that at the optimal breastfeeding duration and that at 24-month breastfeeding duration at high RRRF values, by maternal ART duration

**Supplementary Material 5:** Optimal breastfeeding duration for HIV-exposed infants by RRRF value and maternal ART duration using Spectrum 2016 Data **Supplementary Material 1:** Structure of the CEPAC Infant Model


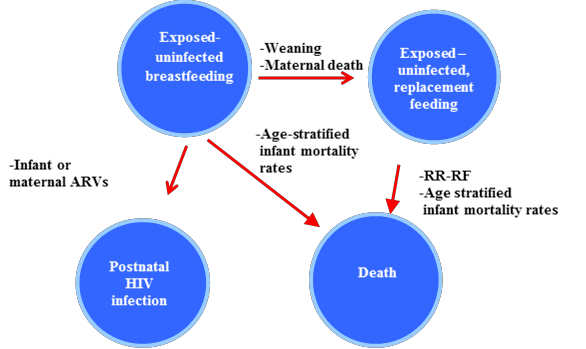


HIV-exposed, uninfected infants enter the CEPAC-Infant model at birth, and are simulated until 24 months after birth. During each month of the simulation, infants face risk of maternal death (this necessarily leading to weaning and an increased risk of infant mortality), infant death, or infant HIV infection through breastfeeding. Risks of each clinical event are stratified by the parameters shown in the figure. The model tallies infant infections and mortality. The primary outcome of the analysis is the proportion of infants alive without HIV infection at 24 months after birth (HFS).

**ARVs**: antiretroviral drugs; **RR-RF**: relative risk of mortality among replacement-fed compared to breastfed infants.

**Supplementary Material 2: HFS Calculations by maternal ART duration**

The following is a description of the calculations used to determine infant/child HIV-free survival after the discontinuation of maternal ART. These calculations are based upon the assumption that once women discontinue ART, the subsequent HFS of their infants at each month is equivalent to that of infants whose mothers are ART-naive (maternal ART duration = 0 mo).

1. First, the proportion of infants of ART naïve mothers (ART duration = 0 months) who either die or became HIV-infected over specified intervals over 24 months is calculated (second column).
2. Each subsequent run varies by maternal ART duration. When a mother discontinues her ART use, it is assumed that the infant of the mother will start to experience the same transmission or mortality risks as the infant of an ART-naïve woman at the corresponding month of life.
   1. For example, the proportion of infants among ART-naïve women who become HIV-infected or die between month 3 and 6 of life is 0.0200. We assume that, in a cohort of women that discontinues ART after 3 months, the 9,602,405 children who are HIV-negative and alive at the end of 3 months start to face the same transmission and mortality risks as infants of ART-naïve women and thus the same proportion either die or become HIV-infected between month 3 and 6 of life (0.020). Thus the number of infants alive and HIV-uninfected at 6 months is 9,602,405* (1-0.020) = 9409798. This population of infants then continues to face the corresponding proportional decline for each time period until 24 months when the simulation ends.

The yellow cells below each column indicate the derivations of HFS using the method described above. Full formulas used for each calculation can also be viewed in the Supplementary Excel Document.

| **Maternal ART Duration -->** |  | 0 | 3 | 6 | 9 | 12 |
| --- | --- | --- | --- | --- | --- | --- |
| **Month of Life** | Proportion of infants who are HIV-infected or who have died |  |  |  |  |  |
| 1 | 0.0159891 | 9840109 | 9865891 | 9865891 | 9865891 | 9865891 |
| 3 | 0.032278707 | 9522483 | 9602405 | 9602405 | 9602405 | 9602405 |
| 6 | 0.020058214 | 9331479 | 9409798 | 9480679 | 9480679 | 9480679 |
| 9 | 0.043292816 | 8927493 | 9002421 | 9070234 | 9359682 | 9359682 |
| 12 | 0.042711095 | 8546190 | 8617918 | 8682834 | 8959920 | 9241370 |
| 15 | 0.006025258 | 8494697 | 8565993 | 8630518 | 8905934 | 9185688 |
| 18 | 0.006044595 | 8443350 | 8514215 | 8578350 | 8852101 | 9130165 |
| 21 | 0.00605814 | 8392199 | 8462635 | 8526381 | 8798474 | 9074853 |
| 24 | 0.006089465 | 8341095 | 8411102 | 8474460 | 8744896 | 9019592 |

**Supplementary Material 3:** 24-month HIV-free survival as breastfeeding duration, maternal ART duration, and RRRF are varied*

**
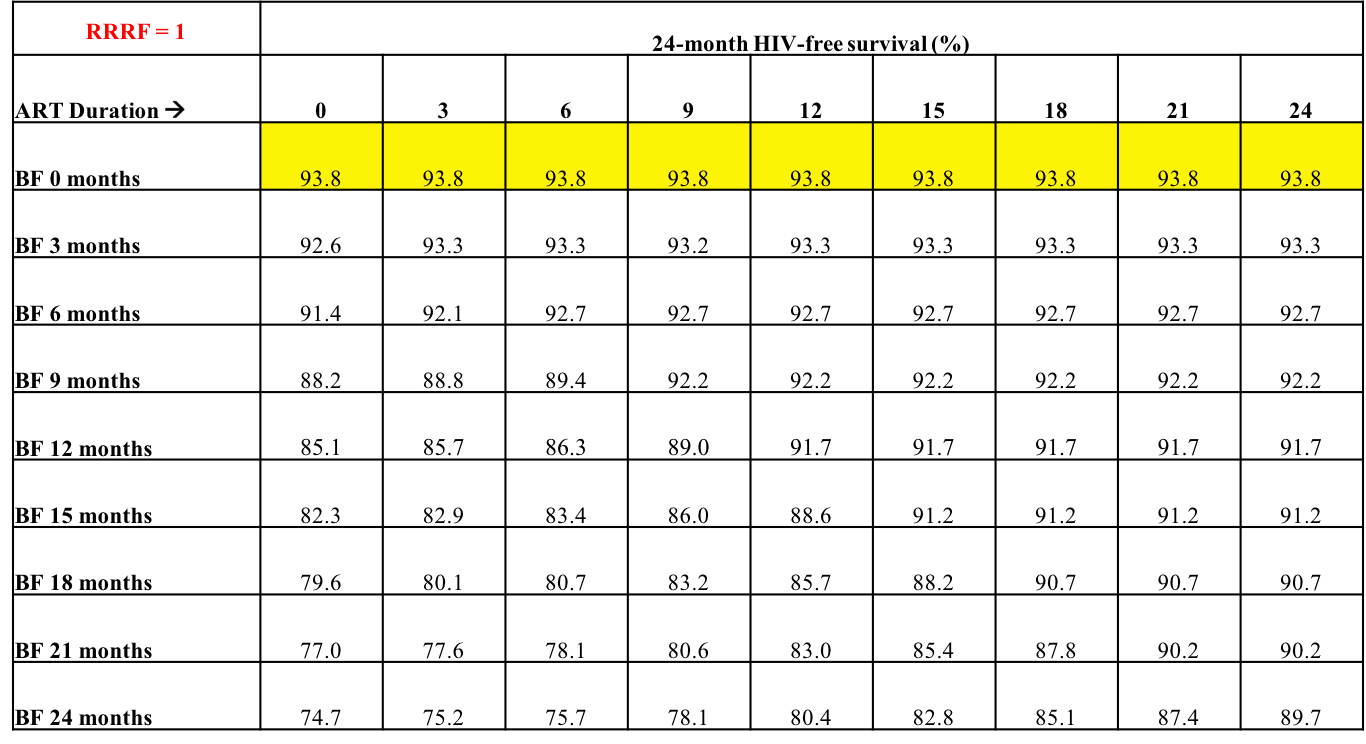
**

**
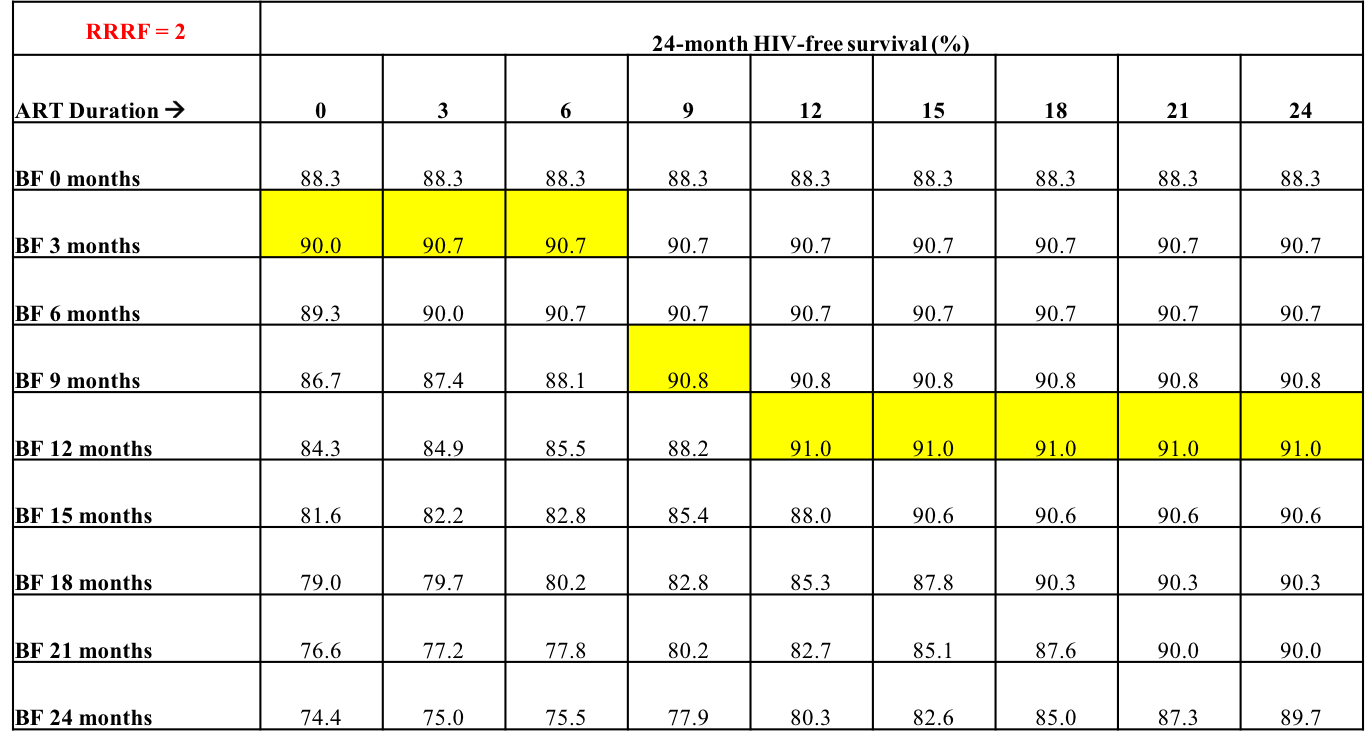
**

*Yellow highlighting indicates the optimal breastfeeding duration for each ARV duration

**ART**: antiretroviral therapy; **BF**: breastfeeding duration; **RRRF**: relative risk of replacement feeding

**Supplementary Material 3:** 24-month HIV-free survival as breastfeeding duration, maternal ART duration, and RRRF are varied* (continued)

**
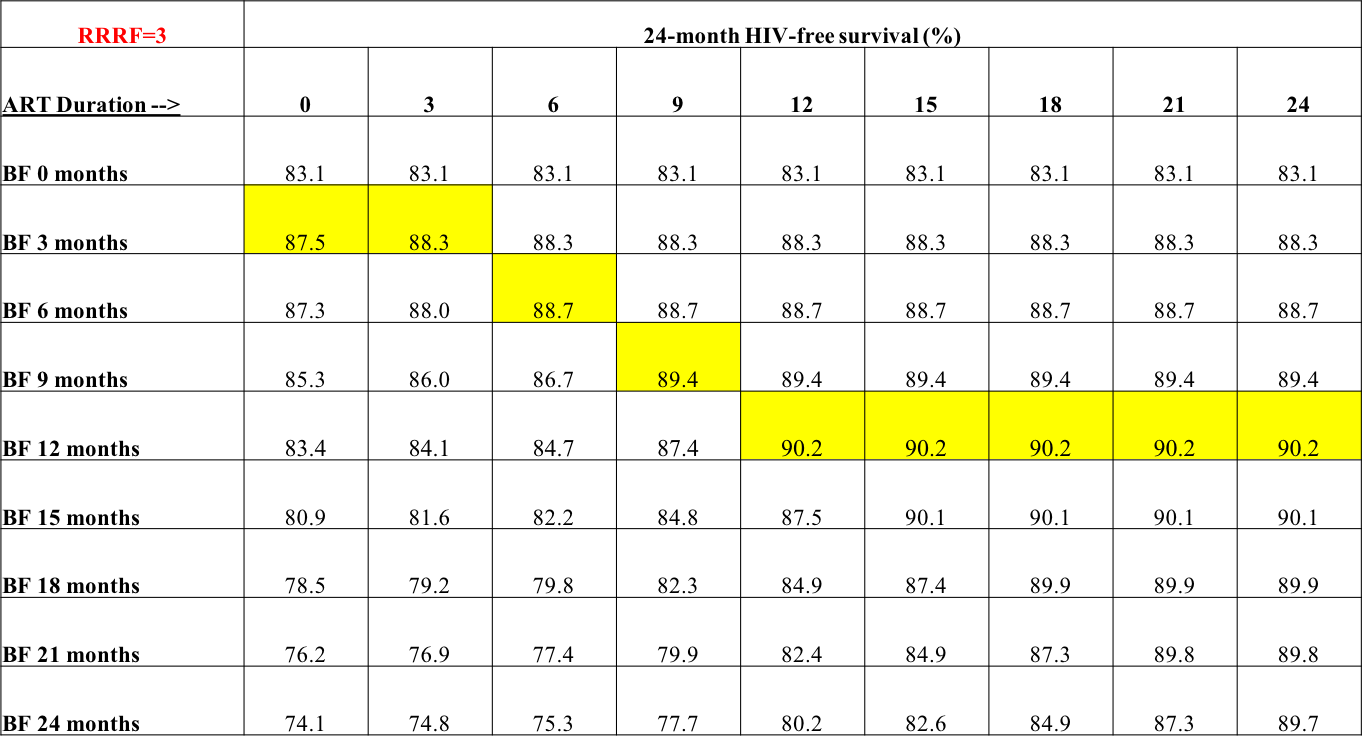
**

**
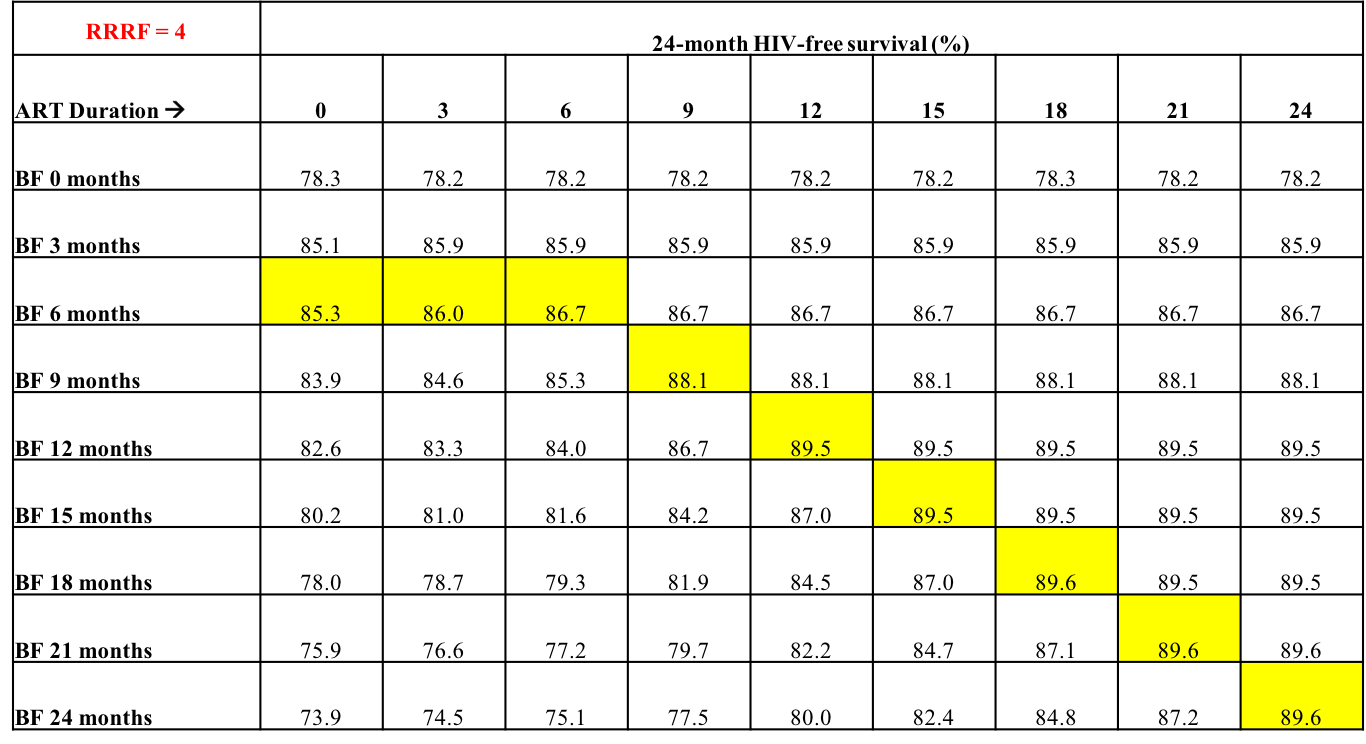
**

*Yellow highlighting indicates the optimal breastfeeding duration for each ARV duration

**ART**: antiretroviral therapy; **BF**: breastfeeding duration; **RRRF**: relative risk of replacement feeding

**Supplementary Material 3:** 24-month HIV-free survival as breastfeeding duration, maternal ART duration, and RRRF are varied* (continued)

**
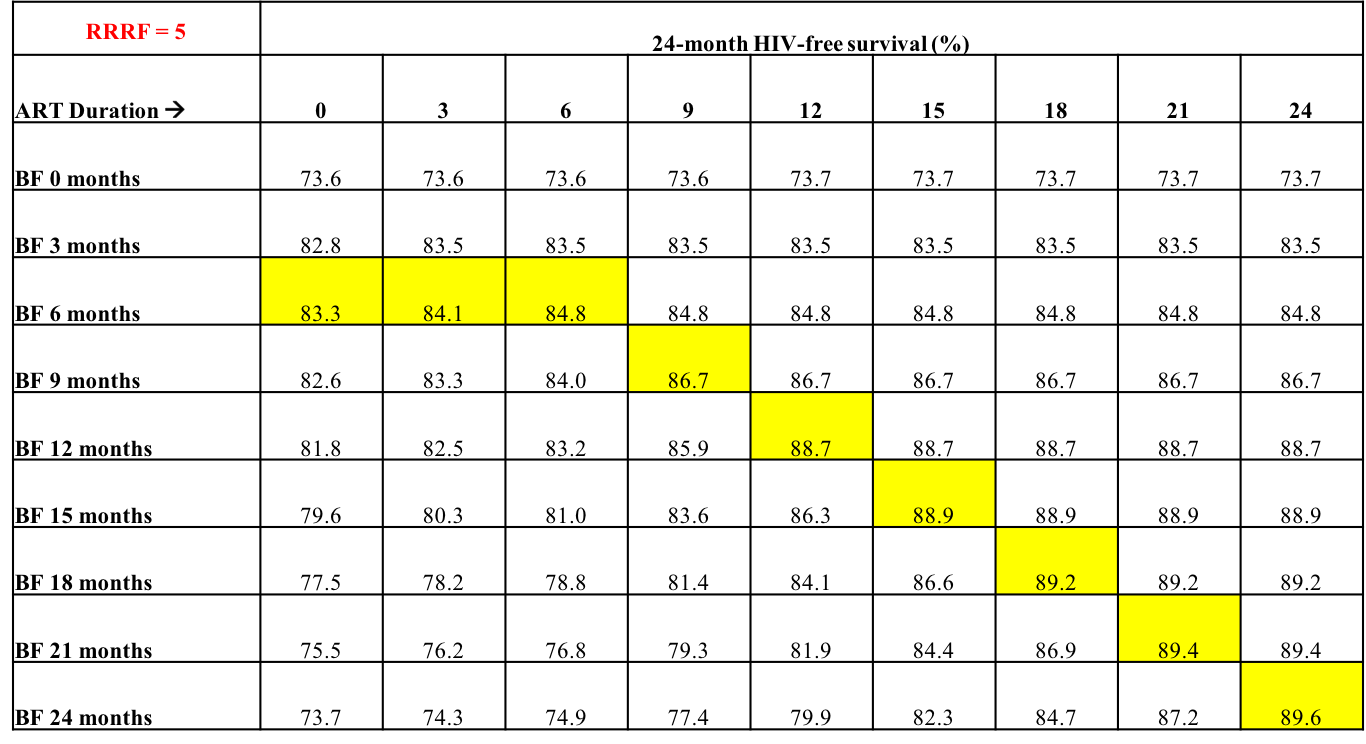
**

**
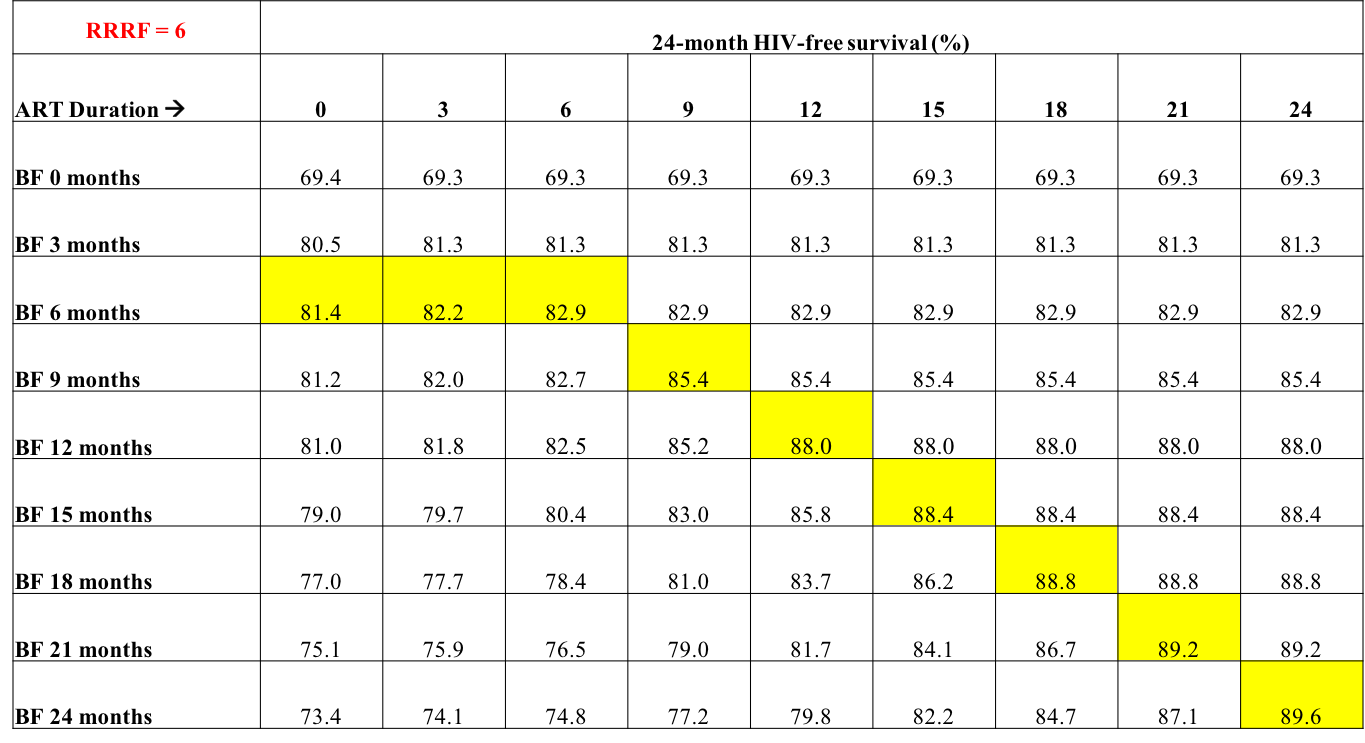
**

*Yellow highlighting indicates the optimal breastfeeding duration for each ARV duration

**ART**: antiretroviral therapy; **BF**: breastfeeding duration; **RRRF**: relative risk of replacement feeding

**Supplementary Material 3:** 24-month HIV-free survival as breastfeeding duration, maternal ART duration, and RRRF are varied* (continued)

**
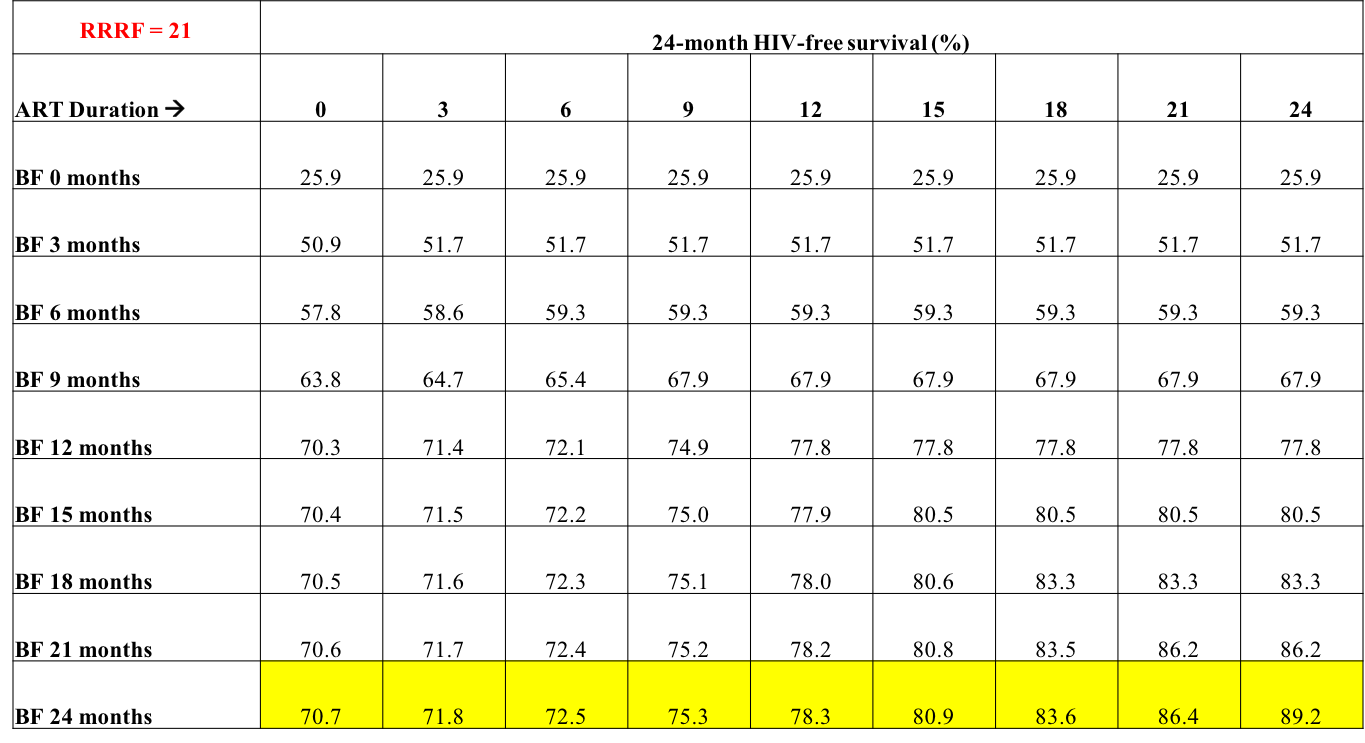
**

*Yellow highlighting indicates the optimal breastfeeding duration for each ARV duration

**ART**: antiretroviral therapy; **BF**: breastfeeding duration; **RRRF**: relative risk of replacement feeding

**Supplementary Material 4A:**  24-month HIV-free survival at the optimal breastfeeding duration at high RRRF values, by maternal ART duration


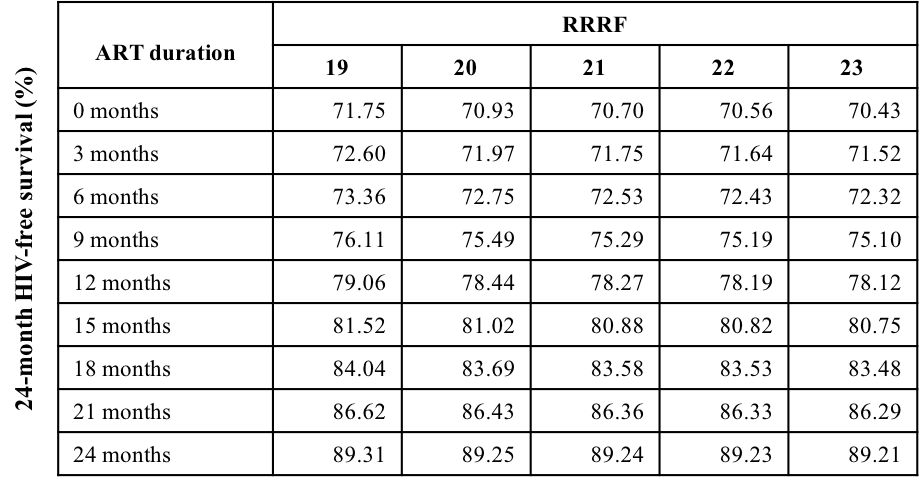


**Supplementary Material 4B**: Optimal breastfeeding durations (indicated by shading) at high RRRF values, by maternal ART duration


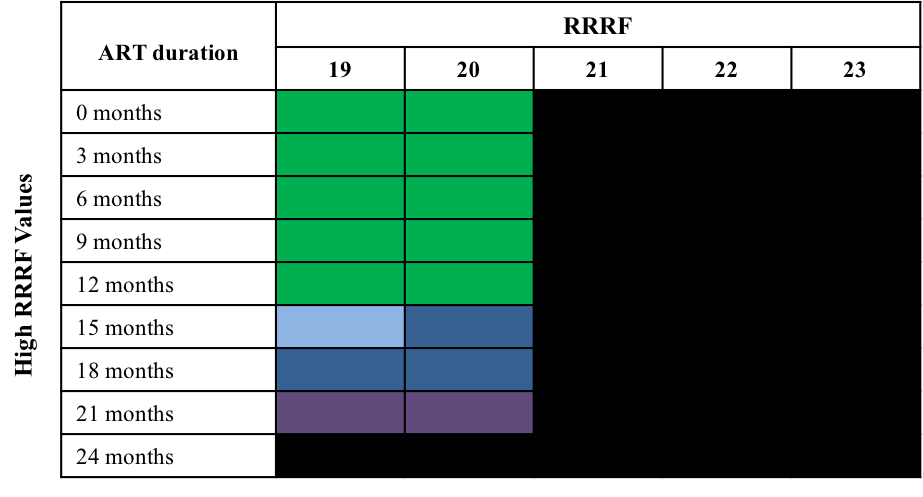


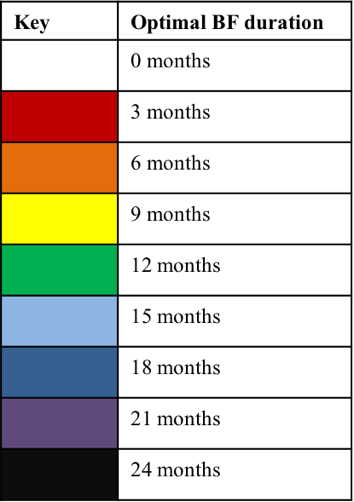


**Supplementary Material 4C:** Difference in 24-month HIV-free survival between that at the optimal breastfeeding duration and that at 24-month breastfeeding duration at high RRRF values, by maternal ART duration


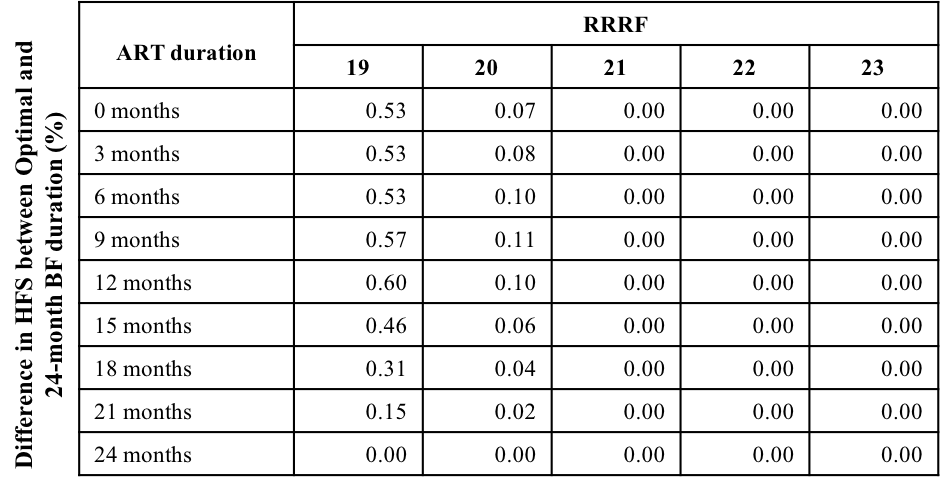


**Supplementary Material 5:** Optimal breastfeeding duration for HIV-exposed infants by RRRF value and maternal ART duration using Spectrum 2016 Data


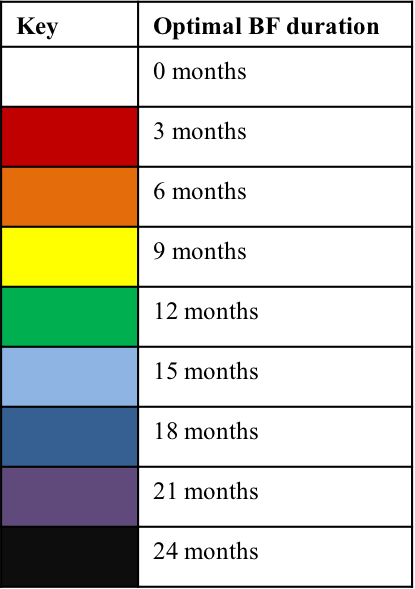


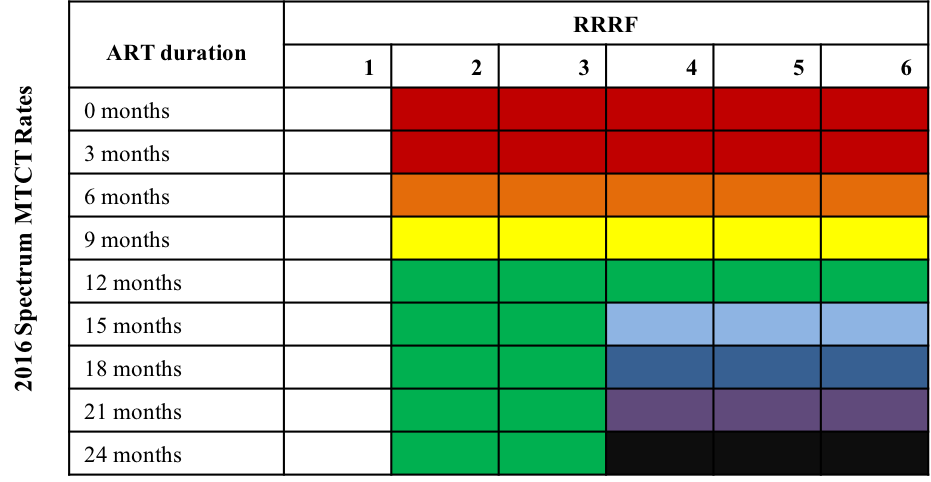

Supplement: Supplementary file 1 — Appendix S1. Additional methods, tables, and figures. [file JIA2-21-e25107-s001.docx]
